# High-Wavenumber Raman Spectroscopy for the Identification and Biochemical Characterization of Microbial Species

**DOI:** 10.1021/acs.analchem.5c05031

**Published:** 2025-12-17

**Authors:** Alec B. Walter, Ezekiel Haugen, Anna S. Rourke-Funderburg, Andrea K. Locke

**Affiliations:** † Vanderbilt Biophotonics Center, 5718 Vanderbilt University, Nashville, Tennessee 37240, United States; ‡ Department of Biomedical Engineering, 5718 Vanderbilt University, Nashville, Tennessee 37240, United States; § Department of Chemistry, 5718 Vanderbilt University, Nashville, Tennessee 37240, United States

## Abstract

Raman spectroscopy is a powerful tool for microbiological and infectious disease research, enabling rapid discrimination of microbial species. While spectral discrimination has typically been performed using the fingerprint region (400–1800 cm^–1^), high autofluorescence backgrounds can degrade signal quality and decrease the overall effectiveness. This work investigates the effectiveness of utilizing the low-background high-wavenumber region (2800–3800 cm^–1^) to both identify and biochemically characterize microbial species. High-wavenumber spectra of 14 microbial species were collected and used to train and validate a multitiered classification model capable of identifying cell wall type (100%), genus (98.9%), and species (97.4%) with high accuracy. Additionally, utilizing a spectral unmixing approach, the relative Raman contributions from proteins, carbohydrates, nucleic acids, lipids, and cell wall components were determined for each species, with general trends matching reported physiological differences. Utilizing a method of converting high-wavenumber Raman spectral fractions to relative dry mass, a biochemical characterization of each species was obtained, with the Raman dry mass characterization of *Escherichia coli* (*E. coli*) closely matching previously reported values. Taken together, these results demonstrate that high-wavenumber Raman spectroscopy is a feature-rich technique capable of performing both accurate discrimination and nondestructive biochemical characterization of microbial species.

## Graphical abstract

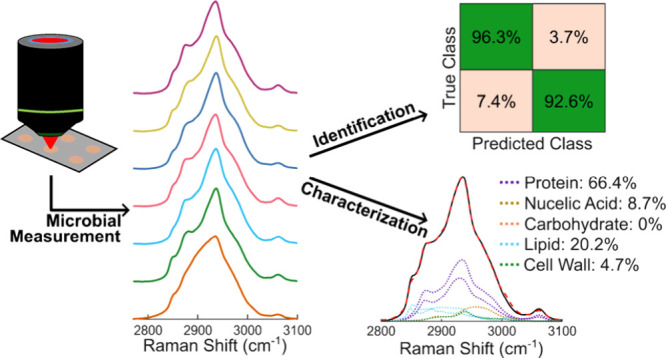

## Introduction

Rapid identification of unknown pathogens is a critical requirement for clinical microbiology and the treatment of infectious diseases. In current practice, both culture-based and molecular diagnostic methods are utilized for pathogen detection due to their proven sensitivity. While the development and utilization of these methods have improved diagnostic outcomes, neither completely meets desired standards. Culture-based methods are rooted in traditional microbiological techniques and provide a cost-effective solution at the expense of time. However, most clinically relevant pathogens require 24 to 72 h of isolation and cultivation before a diagnosis can be made, with extreme cases, such as the causative agent of tuberculosis, *Mycobacterium tuberculosis*, requiring between 2 and 3 weeks of culturing.^1,2^ In many cases, a faster diagnosis is desired to enable the use of targeted therapies, avoiding reliance on broad-spectrum therapeutics. Thus, the capability for rapid and accurate diagnosis serves to minimize the immediate risk to the patient while also helping to diminish the development of multidrug-resistant strains.^3^ Additionally, culture-based methods do not work for the large number of unculturable species that exist across a range of diseases, indicating a need for culture-independent methods of diagnosis. Culture-free molecular techniques, of which polymerase chain reaction (PCR) is the most common, have served to dramatically reduce diagnostic wait times to only a few hours.^1,4,5^ While these techniques can work well in a clinical setting, they are often expensive, requiring continuous replacement of expensive reagents along with specialized equipment and skilled operators. Additionally, a priori knowledge of species-specific genomic targets is required to develop detection protocols, effectively limiting the range of detectable species. As such, there is a growing need for new methods of microbial identification that are rapid, robust, and straightforward to use.

Raman spectroscopy is a vibrational spectroscopy technique that leverages the inelastic scattering of light to provide information on the molecular composition of a sample. Raman spectroscopy has been shown to be a powerful tool for microbiological and infectious disease applications, enabling rapid discrimination of pathogens across a range of diseases including urinary tract infections, middle-ear infections, and meningitis.^6–17^ For these microbiological studies and biomedical applications in general, Raman spectroscopy has traditionally focused on the fingerprint region (400–1800 cm^–1^) of the spectrum as it contains multiple discrete peaks that can be attributed to different biomolecules.^12,13^ In contrast, the high-wavenumber region (2800–3800 cm^–1^) contains only two major Raman bands representative of Oxygen–Hydrogen bonds, which are dominated by water, and Carbon–Hydrogen (−CH_*x*_) bonds, which are present in all major biomolecules. Due to the ubiquitousness of these bonds in biological samples and the high degree of spectral overlap within the bands, the high-wavenumber region is often left unutilized. Nevertheless, the high-wavenumber region has its own advantages, such as significantly higher Raman intensities compared to the autofluorescence background, which have enabled it to provide diagnostic accuracies on par with the fingerprint region for several noncommunicable diseases.^18–20^ This reduction in the fluorescence background is expected to be especially impactful for microbiological work due to the high autofluorescence of many bacterial species in the fingerprint region, as seen in Figure S1. While some microbial Raman studies have included the −CH_*x*_ band with the fingerprint region, they typically have utilized broad spectral ranges that simultaneously capture both regions at the expense of spectral resolution.^7,8,21^ There have so far been no studies evaluating the effectiveness of utilizing only high-resolution, high-wavenumber spectra for microbiological characterization.

This work investigates the hypothesis that high-wavenumber Raman spectroscopy is a feature-rich technique capable of discriminating and characterizing microbial species when used alone. To test this hypothesis, high-wavenumber spectra of 14 pathogenic and commensal microbial species were collected and used to train and validate a multitiered classification model. Additionally, spectral unmixing analysis was performed, using basis spectra from 11 pure biomolecules representative of microbial composition, to determine the ability of high-wavenumber Raman spectroscopy to provide accurate biochemical characterization of microbiological samples. To obtain a more physiologically relevant characterization, a new method is proposed that leverages the inherent nature of the −CH_*x*_ band to derive the relative mass fractions from the Raman spectral fractions. Validation of this method was performed using reported values for the dry mass composition of *E. coli.*

## Experimental Section

### Microbial Species and Culturing

A total of 13 bacterial species and one yeast species were used in this work, with a detailed list of the species, strains, and utilized growth media found in Table S1. All species were sourced from the American Type Culture Collection (ATCC) except for the USA300 LAC strain of *S. aureus*.^22^ The yeast species was included in this work to establish the ability to distinguish bacteria from other pathogenic and commensal microbial species. *Candida albicans* was chosen as the representative species as it is a common commensal organism in the human gastrointestinal tract and is often used as a model organism for fungal pathogens.^23^ Each species was initially grown from frozen stock on solid media overnight at 37 °C and 5% CO_2_, except for *C. albicans,* which was grown at 30 °C in air. Monoclonal liquid cultures were made by inoculating single colonies from these cultures into 3 mL of liquid media and growing them overnight. *S. aureus*, *S. epidermidis*, *E. coli*, and *P. aeruginosa* were cultured in tryptic soy broth at 37 °C in air, while *C. albicans* was grown in Sabouraud dextrose broth at 30 °C to ensure minimal hyphae formation. To maintain sufficient aeration for these species, a shaking incubator (MaxQ 4450, Thermo Scientific) was used with an orbital speed of 294 rpm. *S. mutans*, *S. pneumoniae*, and *S. agalactiae* were grown without aeration in brain heart infusion at 37 °C and 5% CO_2_, while *H. influenzae* was grown in similar conditions in a supplemented brain heart infusion broth containing 0.01 mg/mL hemin and 0.02 mg/mL nicotinamide adenine dinucleotide. *L. crispatus*, *L. iners*, and *G. vaginalis* were grown under the same conditions but using De Man–Rogosa–Sharpe (MRS) media for *L. crispatus* and NYCIII media for *L. iners* and *G. vaginalis*. Finally, *M. tuberculosis* and *M. bovis*, were not grown on solid media like the other species. Instead, liquid cultures were made by diluting 0.5 mL of frozen primary culture into 9.5 mL of fresh Middlebrook media supplemented with 10% albumin-dextrose-catalase growth supplement, 0.2% glycerol, and 0.1% Tween-80. These cultures were grown on a rolling incubator for 7 days at 37 °C and 5% CO_2_ before use.

### High-wavenumber Raman Measurements

To prepare the microbial species for measurement, the growth media was washed from the planktonic bacteria. This was accomplished by removing 1 mL of liquid culture and centrifuging it at 3300 rcf for 8 min, discarding the supernatant, and resuspending the pellet in sterile phosphate-buffered saline. Washing was performed twice before the sample was centrifuged a third time to isolate the cellular pellet. 1 μL droplets of the pellet were deposited onto a microscope slide coated in standard aluminum foil (Reynolds Wrap, Reynolds) and allowed to dry.

High-wavenumber Raman measurements of the dried samples were acquired using a Renishaw inVia Raman microscope using a 785 nm excitation laser and a 1200 lines/mm grating, which provided a spectral resolution ∼ 1 cm^–1^ across the full measurement range of 2760 cm^–1^ to 3500 cm^–1^. The relative wavenumber axis for the system was calibrated daily using a silicon standard, while spectral response calibration was performed using a 785 nm luminescence standard reference material (SRM 2242, NIST). Individual sample spectra were taken under a 50*x*/0.75NA objective, which supplied approximately 80 mW of power at the sample, measured both before and after measurement sessions. The measurement spot size was approximately 4.5 μm in diameter. A total integration time of 50 s (10-s exposure, 5 accumulations) was used to compensate for the reduced quantum efficiency of the detector at the measurement wavelengths. For each culture, three different droplets were measured, with 3 spectra being obtained from each droplet. Measurements were performed along the edge of the dried spots where the cells were only a few layers thick to reduce out-of-focus signal collection. The combination of taking multiple separate measurements and measuring groups of cells helped to ensure that the acquired spectra accounted for the overall biochemical composition of the microbial species. This process was repeated three times for each utilized species, using separately grown cultures each time to ensure a degree of population variance, resulting in 27 total spectra per species. The triplicate cultures for each species were grown and measured on separate days to better ensure the technical reproducibility of the measurements.

### Spectral Preprocessing

All acquired Raman spectra were preprocessed using previously developed methodologies performed within the MATLAB 2024a environment.^20^ The collected spectra were first truncated from 2765 cm^–1^ to 3495 cm^–1^ before a baseline correction step was performed to remove the autofluorescence background. This was accomplished using a second order modified polynomial fit, excluding the wavenumber region between 2840 and 2990 cm^–1^ to prevent the relatively broad −CH_*x*_ band from influencing the background estimation.^20,24^ The resulting Raman spectra were smoothed using a second-order Savitsky-Golay filter with a window size of 11, corresponding to an average spectral window 8.25 cm^–1^, before being binned to a 1 cm^–1^ bin-width. To isolate the −CH_*x*_ band, a final truncation was performed between 2770 cm^–1^ and 3100 cm^–1^. For visualization and analysis purposes, spectra were mean-normalized. Spectra for each species were averaged together and the 95%/95% tolerance interval for each species was determined. Given the number of independent samples and the total number of spectra per species, this interval was determined to be approximately ± 2.31 times the standard deviation.

### Classification Model

To assess the ability of the −CH_*x*_ band to distinguish and classify different microbial species, a binary decision tree approach was used with the structure and hyperparameters shown in Scheme 1. Each node of the tree utilized partial least-squares (PLS) for supervised dimensionality reduction, followed by a support vector machine (SVM) classification model using a Gaussian kernel. The number of PLS latent variables (LVs) utilized by the SVM was optimized for each node, with no node requiring more than 5 total LVs. The gamma parameter for the SVM kernels was also optimized for each node, with values ranging from 0.49 to 9.95.

**Scheme 1:**
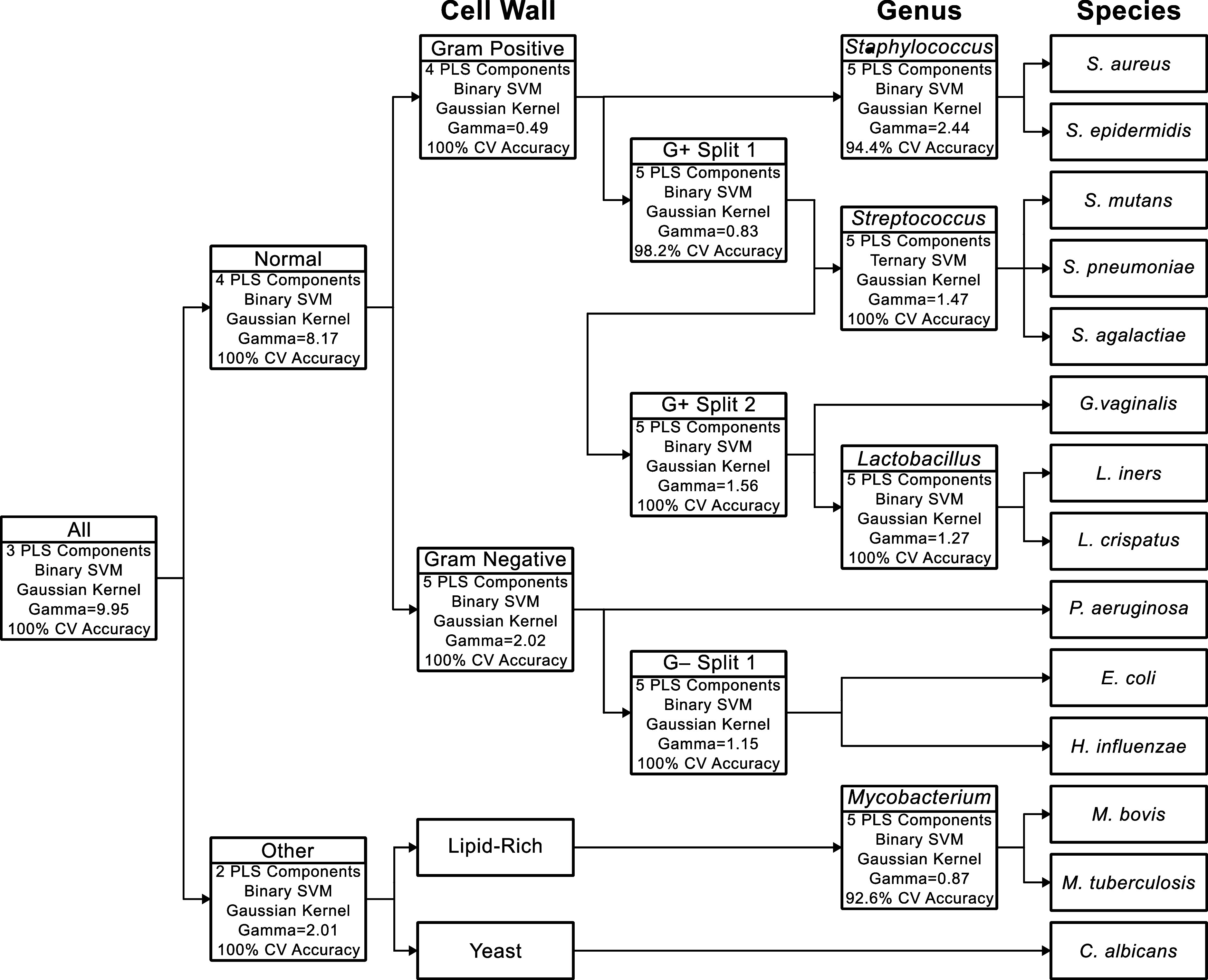
Structure of the Binary Decision Tree Model. Each Decision Node Represents a Separate PLS-SVM Model, Indicated by the Inclusion of the Optimized Hyperparameters and Cross-Validation Accuracy

To assess the performance of the decision tree, cross-validation was performed using a leave-one-culture-out (LOCO) approach, where all nine spectra from a given culture were held back together, to mitigate bias in the model. Leveraging the decision tree structure, the prediction accuracy was determined for three different levels of specificity: cell wall, genus, and species. Cell wall type, representing the coarsest level of differentiation, was defined as either Gram-positive, Gram-negative, lipid-rich, or yeast. Yeast species have distinct cell walls, as compared to bacteria, which are largely composed of glucans and chitin.^25^ Bacteria with lipid-rich cell walls, namely the *Mycobacterium* species used here, were separated from the other species as their high concentrations of cell wall lipids significantly alter their overall biochemical composition.^26^ At the genus level, the only genera that were classified were those for which more than one species were included in this work, encompassing *Staphylococcus*, *Streptococcus, Mycobacterium*, and *Lactobacillus*. At the final classification level, spectra were categorized as one of the 14 utilized species.

### Spectral Unmixing and Biochemical Characterization

To approximate the biochemical composition of the different microbial species, five major categories of biomolecules were chosen (proteins, nucleic acids, carbohydrates, lipids, and cell wall components), which were represented using eight different pure components. Histone (H9250, Sigma-Aldrich) was chosen to represent histone-like proteins, which are one of the more prevalent categories of bacterial proteins, while bovine serum albumin (A2153, Sigma-Aldrich) was used to approximate the general protein population.^27,28^ Glycogen (G0855, Sigma-Aldrich) was selected as the representative carbohydrate, while nucleic acids were represented using DNA (D1501, Sigma-Aldrich). For lipids, phosphatidylethanolamine (PE; P1223, Sigma-Aldrich), the most abundant lipid in *E. coli,* and oleic acid (364525, Sigma-Aldrich) were used to represent solid and liquid microbial lipids, respectively.^29^ While oleic acid itself is not a microbial lipid, it has been demonstrated in previous works as an accurate model for generic biological lipids in the high-wavenumber region.^20,30^ To account for the cell walls of the bacteria, which can make up a significant proportion of their total mass, purified peptidoglycan was used (69554, Sigma-Aldrich).^31^ Additionally, to both account for varying cross-linking percentages between species and the potential of cross-talk between the pure proteins and the high amino acid content of peptidoglycan, N-acetylmuramic acid (MurNAc; A3007, Sigma-Aldrich), one of the amino sugars forming the peptidoglycan backbone, was included independently.^31^ Taken together, the selected components are expected to act as general representatives of the biomolecules found in microbial species. Finally, to account for the unique lipid inclusions in the cell wall of *Mycobacterium* species, all three major forms of mycolic acids (alpha-MA, 791280; keto-MA, 291281; methoxy-MA, 291282, Avanti Research) were utilized.^26^ The high-wavenumber Raman spectra of the pure components were acquired and processed using the methodology described above. Three separate spectra were taken for each component, with the average of the normalized spectra being used in further analysis.

Spectral unmixing was performed for each microbial species using a non-negative least-squares approach. To better approximate the Raman fractional contributions, both the target and the component spectra were normalized prior to use, such that their respective area-under-the-curves (AUC) were equal to 1. The eight major components described above were used to spectrally unmix each species while the mycolic acids were only utilized for the spectral unmixing of the *Mycobacterium* species. After unmixing, individual component contributions were summed together to yield Raman fractions for the component classes: protein, nucleic acid, carbohydrate, lipid, cell wall, and mycolic acid. To help prevent any noise that may be present in individual spectra from negatively influencing the results of the spectral unmixing, analysis was performed on the average spectra for each droplet, resulting in 9 unmixed spectra per species. From this, the average Raman fractions for each species and the corresponding standard error of the mean (SEM) were obtained.

### Raman Dry Mass

While the resulting Raman fractions are indicative of the composition of the bacteria, they do not directly match the physical concentrations of each component. This is because the different biomolecular classes have different Raman cross sections.^32,33^ The Raman cross-section for a given vibrational mode is primarily dependent on the polarizability tensor and the atomic prevalence of the specific molecular bond.^32^ This relationship is typically not beneficial when analyzing fingerprint Raman spectra, as each vibrational mode will have a different, potentially unknown, polarizability. However, as the high wavenumber spectra collected in this work consists of only the −CH_*x*_ stretching modes, an assumption can be made that the polarizabilities are approximately equal and that differences between biomolecules are predominantly due to the relative prevalences of their −CH_*x*_ bonds.

From this, a relationship for the relative Raman contribution of a biomolecule class can be approximated as

$$\frac{{AUC}_{X}}{{AUC}_{CH}}=F_{X}\approx \frac{N_{X}}{N_{total}}$$ 1

where *F*_*X*_ is the Raman spectral fraction provided by biomolecule X, *N*_*X*_ is the number of −CH_*x*_ bonds originating from that biomolecule, and *N*_*total*_ is the total number of those bonds present in the sample. While the absolute values for the number of −CH_*x*_ in the sample volume are difficult to know, the relative amount per mass can be estimated as

$$N=B\times \frac{M}{MW}$$ 2

where M is the total mass, MW is the molecular weight, and B is the number of −CH_*x*_ per molecule, determined as the number of carbon atoms with at least one covalent bond to a hydrogen. To simplify the wide range of values that MW and B can take for different biomolecules within the same class, estimates were derived using a monomer-based approach, with the utilized values being found in Table S2. For proteins, values for an average amino acid were determined using the reported amino acid composition of *E. coli*.^34,35^ Carbohydrates were approximated as consisting of only glucose, while the values for nucleic acids were determined using the DNA nucleotides, for which the predominant source of −CH_*x*_ bonds is the deoxyribose sugar.^30^ For the cell wall components, peptidoglycan and the amino sugar were treated separately, with both utilizing a similar weighted average approach. The values for the amino sugar were determined using an equal representation from N-acetylglucosamine and N-acetylmuramic acid, while peptidoglycan used an average of the two amino sugars to the common peptidoglycan pentapeptide Ala-Glu-Lys-Ala-Ala.^31^ Finally, the values for the lipids were approximated using a 16:0/18:1 phosphatidylethanolamine (PE) and the mycolic acids were represented using alpha-mycolic acid, which has been reported to be the most common mycolic acid produced by *Mycobacterium* species.^36^

Combining eqs 1 and 2 yields an equation for each biomolecule class which takes the form

$$F_{X}=\frac{B_{X}\times \frac{M_{X}}{{MW}_{X}}}{B_{P}\times \frac{M_{P}}{{MW}_{P}}+B_{N}\times \frac{M_{N}}{{MW}_{N}}+B_{C}\times \frac{M_{C}}{{MW}_{C}}+B_{L}\times \frac{M_{L}}{{MW}_{L}}+B_{W}\times \frac{M_{W}}{{MW}_{W}}+B_{A}\times \frac{M_{A}}{{MW}_{A}}+B_{M}\times \frac{M_{M}}{{MW}_{M}}}$$ 3

where the subscripts P, N, C, L, W, A, and M denote protein, nucleic acid, carbohydrate, lipid, peptidoglycan, amino sugar, and mycolic acid, respectively, and the subscript X represents the biomolecule the equation is for. Given knowledge of the overall dry mass of the measurement volume, the dry mass of each component, here on referred to as the Raman dry mass, can be determined by solving the system of equations. When the true mass of the measured region is difficult to measure or unknown, such as in this work, the relative Raman dry mass composition can instead be determined by applying the constraint that the M values sum to 100%. Using this process, the Raman dry mass composition was determined for all 14 species, and the results for *E. coli* were compared to the biomolecular composition values reported in the literature for the species.^34,35,37^

## Results and Discussion

### High-Wavenumber Microbial Characterization

The measured −CH_*x*_ stretching bands for the 14 microbial species are shown in Figure 1. Using the described procedures for sample preparation and spectral acquisition yielded highly consistent spectra across all measurements, with the average coefficient of variation being only 2.1%, with a range from 0.98% (*P. aeruginosa*) to 3.5% (*S. pneumoniae*). This consistency indicates that any observed spectral differences can be attributed to changes in the underlying composition of a microbial sample. Additionally, the small spectral differences observed across the three cultures indicates that there is a high degree of reproducibility for the high-wavenumber Raman measurements.

**Figure 1:**
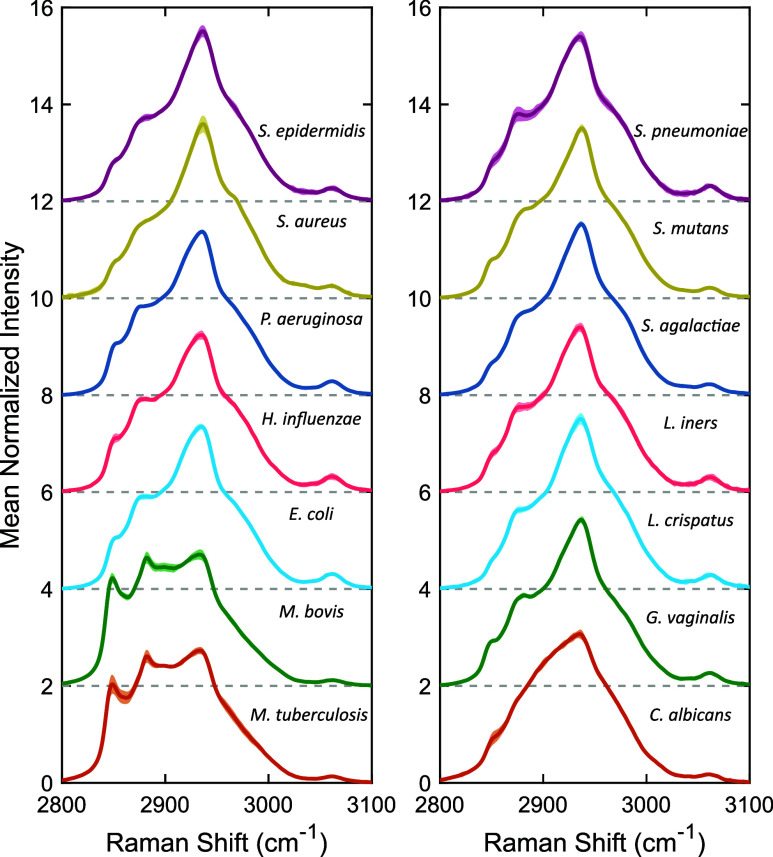
High-wavenumber Raman spectra for the 14 microbial species. Each spectrum represents the mean (solid line) and 95%/95% tolerance interval (shaded area) for the 27 measurements. Spectra are mean-normalized and vertically offset, as indicated by the gray lines.

Tentative peak assignments for the vibrational modes contributing toward the high-wavenumber spectra of the microbial species can be found in Table 1. Overall, the spectra for all species were found to be dominated by the 2935 cm^–1^ peak, which is commonly associated with protein and amino acid content.^38,39^ Additionally, all species contained either a peak or shoulder at 2850 cm^–1^, which is characteristic of lipids.^38,40^ This peak, along with the associated 2882 cm^–1^ peak, are especially prominent for the *Mycobacterium* species, providing them a distinct spectral shape as compared to the other species. These differences are likely due to the presence of mycolic acids, which are long-chain fatty acids that these species incorporate into their cell walls.^26^ Another species that stands out spectrally due to its cell wall composition is *C. albicans*. As a yeast, the cell wall of *C. albicans* contains the polysaccharides glucan and chitin.^25^ Polysaccharides exhibit a characteristic peak at around 2910 cm^–1^, which, when combined with the protein peak, contributes to the observed smooth and broad spectral shape of *C. albicans*.^41^

**Table 1:** Tentative Peak Assignments for the High-Wavenumber Spectral Features Observed in the Microbial Samples

**Wavenumber (cm** ^**–1**^ **)**	**Vibrational Assignment**	**Tentative Associated Biomolecules**
2845–2855	–CH_2_ symmetric	Lipids
2870–2900	–CH_2_ asymmetric	Lipids, proteins
2910–2930	–CH	Carbohydrates, lipids
2930–2940	–CH_3_ symmetric	Proteins
2950–2970	–CH_3_ asymmetric	Proteins
2960–2970	–CH	Nucleic acids, proteins, carbohydrates
3000–3010	=CH	Unsaturated lipids
3055–3065	=CH aromatic	Proteins

While more subtle in comparison, distinct differences can be observed both between and among the remaining bacterial species. The 2935 cm^–1^ peak is observed to be broader and more rounded for the Gram-negative species as compared to the Gram-positive. This is matched by an increased prominence of the 3062 cm^–1^ peak, which has been attributed to the =CH bonds of aromatic amino acids. Taken together, this may indicate there is a difference in both the total protein content and the amino acid composition between the two groups. Another observed difference is the relative prominence of the 2850 cm^–1^ lipid peak. On average, Gram-negative species were found to have a stronger lipid signal, which could be due to them having both an inner and outer lipid bilayer, as opposed to the single lipid membrane of Gram-positive bacteria.^31^ Overall, while the high-wavenumber region lacks the distinct peaks characteristic of the fingerprint region, the −CH_*x*_ stretching band was found to contain a variety of rich information with detectable differences between species.

### Classification Model

With the observed differences in the −CH_*x*_ stretching band, a PLS-SVM decision tree was created, as outlined in Scheme 1. Using 11 decision nodes, this tree categorizes spectra into four different cell wall types, the four genera with more than one utilized species, and, finally, the 14 individual species. This format for the classification helps to simplify the complexity of multiclass classification into a number of increasingly separated binary classification models.^42^ In addition to simplifying the PLS dimensionality reduction and reducing the number of latent variables utilized for each node, the decision tree structure also prevents significantly different species, such as the yeast *C. albicans*, from affecting the performance of more nuanced classification, such as between related bacterial species. The decision tree can also be readily augmented with new classes, allowing any future works that, for example, investigate a wider range of yeast species to be integrated into the model by expanding upon the respective branch. However, inclusion of genera or species more closely related to the bacteria used in this work would likely require at least a portion of the decision tree structure to be reoptimized.

From the LOCO cross-validation, the accuracies of each node (Scheme 1) and each of the classification tiers were determined. As shown in Figure 2A, the decision tree model achieves 100% accuracy in determining cell wall type. This is likely due to the large spectral differences described above, which originate from the distinct differences in cell wall morphology and composition. For the genus classification (Figure 2B), only three *Streptococcus* spectra were misclassified as *Lactobacillus* out of the 243 genus-associated spectra. This resulted in an overall accuracy of 98.8% and a class-averaged sensitivity and precision of 99.1% and 98.7%, respectively. While only four genera were included in this model, these results indicate that the high-wavenumber region may contain information indicative of phylogenetic classification.

**Figure 2:**
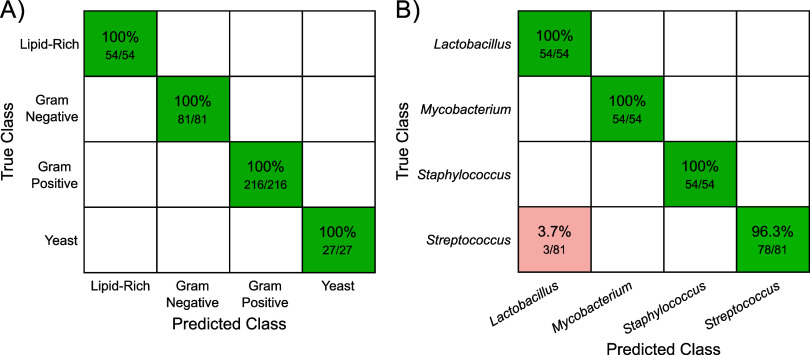
Confusion matrices denoting the leave-one-culture-out cross-validation accuracy of the PLS-SVM decision tree when determining the cell wall (A) and genus (B) classifications.

The confusion matrix for the species classification is found in Figure 3. While this level of granularity led to individual accuracies beginning to decrease, the overall accuracy remained at 97.4%, with species-averaged sensitivities and precisions of 97.4% and 97.6%, respectively. The errors from the genus level propagated into the species classification, resulting in three *S. pneumoniae* spectra being misclassified as *L. crispatus*. The remaining seven misclassified spectra all resulted from errors within genera. For example, of the 54 *Staphylococcus* spectra, two spectra of *S. aureus* and one of *S. epidermidis* were classified as the other species (Figure 3). Additionally, for *Mycobacterium*, 4 of the 27 *M. bovis* measurements were misclassified as *M. tuberculosis,* resulting in the overall lowest class accuracy of 85.2% (Figure 3). While the overall high accuracy of the model was achieved through an initial optimization of the decision tree layout, the number of PLS latent variables, and the SVM hyperparameters, additional optimization would likely serve to further improve the performance. This includes investigating the effect that different methods of dimensionality reduction, such as principal component analysis and Lasso regression, and different types of discriminatory models, such as linear discriminant analysis and convolutional neural nets, have on overall classification accuracy.

**Figure 3:**
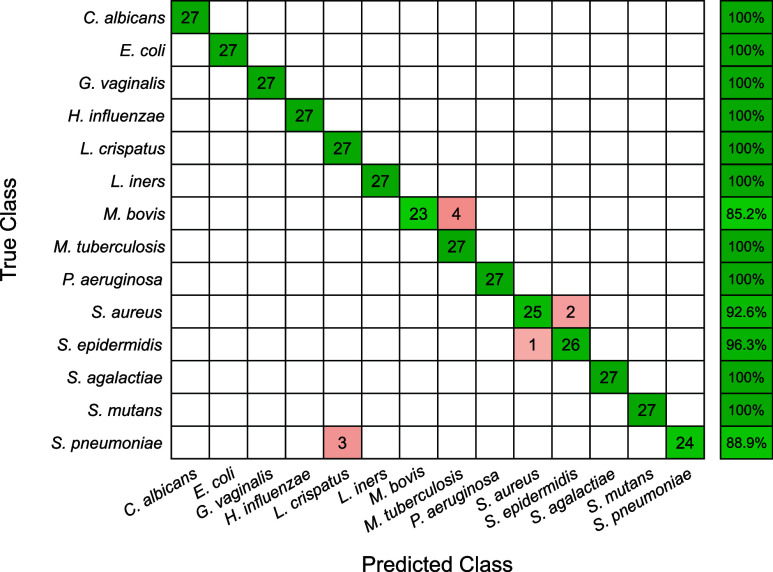
Confusion matrix denoting the leave-one-culture-out cross-validation accuracy for the species-level classification.

Overall, the performance of the high-wavenumber classification model was found to be at or above the performance of other Raman-based classification models.^6,11,14–17^ This is notable, as most previous studies have utilized the “fingerprint” region, assuming it to be more information-rich. However, the comparable performance of this classification model implies that a similar level of biochemical information may be embedded within the −CH_*x*_ band. Additionally, while dedicated studies would need to be performed to definitively determine if the combination of the two spectral regions would further improve classification, existing studies utilizing both regions have reported comparable classification accuracies, potentially indicating that there is overlapping information between the two regions.^18,19^ While this may be due to both regions effectively sampling the same underlying biochemical composition, despite providing information on different bond types and vibrational modes, it may also be due to experimental limitations. Previous studies utilizing both fingerprint and high-wavenumber spectra have utilized spectrographs with wide detection ranges, to simultaneously capture both regions, at the expense of spectral resolution.^7,8,21^ This decrease in the effective spectral information may be the cause of the observed similarities in classification accuracy. As such, future work is needed to determine whether combining separately measured fingerprint and high-wavenumber spectra improves classification accuracy, especially for differentiating between classes with increasingly small spectral differences, such as closely related species or strains of the same species.

### Biochemical Components for Spectral Unmixing

The −CH_*x*_ stretching band spectra for the measured pure biochemical components can be found in Figure 4. While the tentative peak assignments for the vibrational modes that make up this region of the Raman spectra have a high degree of overlap, each class of biomolecule was observed to have a distinctive spectral shape. The proteins, lipids, nucleic acids, and carbohydrates were found to closely match previously reported measurements.^40,41,43^ Glycogen was found to have a broad and smooth spectral shape, likely due to the blending of the discrete vibrational modes found in pure glucose, with a maximum located at 2910 cm^–1^ and a shoulder at 2945 cm^–1^.^41^ With the −CH_*x*_ signal for nucleic acids almost entirely originating from the pentose sugar backbone, a similar blending of vibrational modes is observed for DNA. The broad DNA band centered at 2955 cm^–1^ aligns with the discrete peaks reported for deoxyribose.^41^

**Figure 4:**
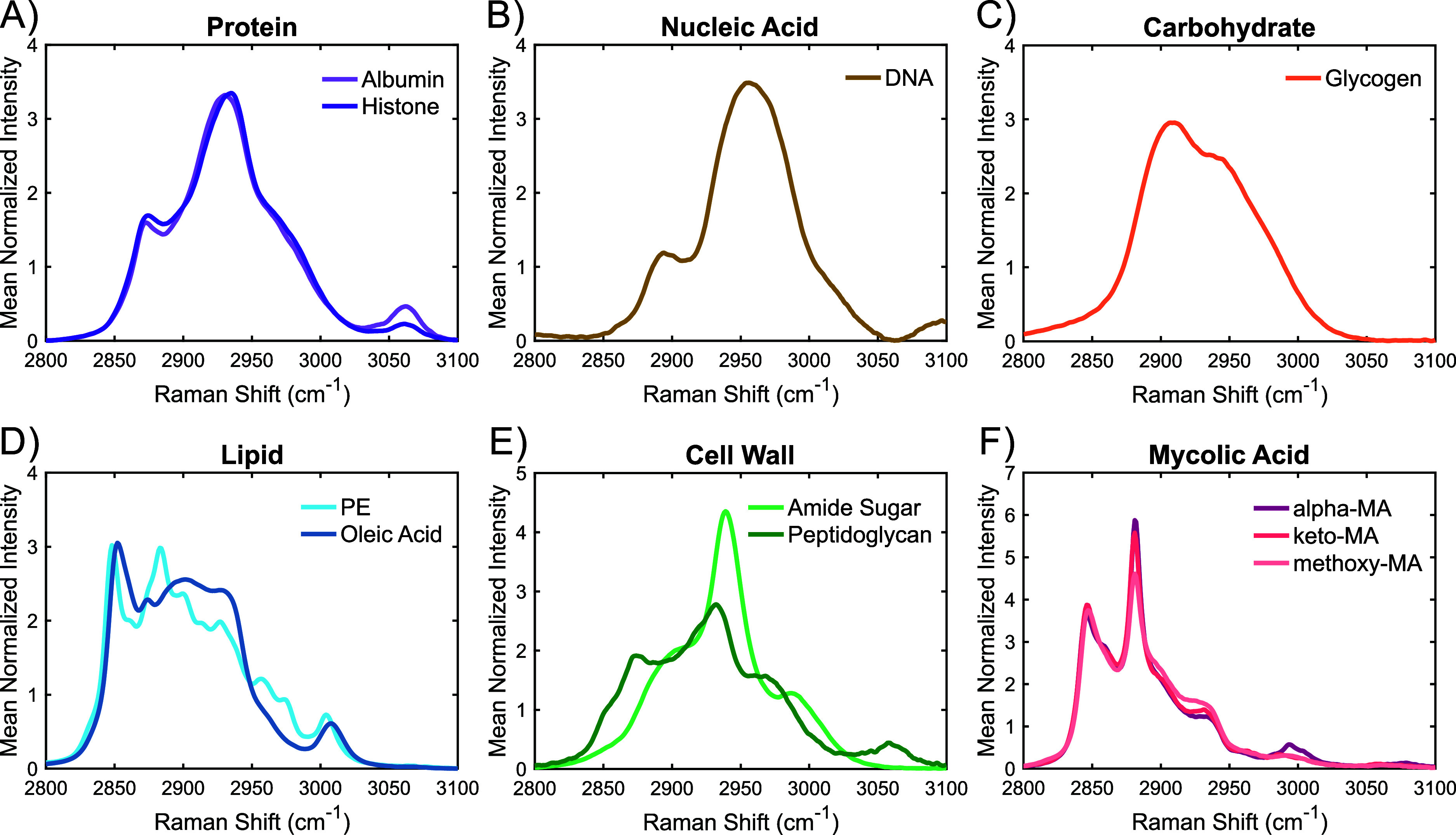
High-wavenumber Raman spectra of the pure components used for spectral unmixing. Components were categorized as proteins (A), nucleic acids (B), carbohydrates (C), lipids (D), cell wall components (E), and mycolic acids (F). Individual spectra are mean-normalized for visualization.

For both the protein and lipid classes, the two individual components used for each were found to be broadly similar with specific spectral distinctions. For the lipids, oleic acid exhibits a broader spectral shape compared to the more well-defined peaks of PE, with both being dominated by the symmetric and asymmetric stretching modes of the −CH_2_ group. Additionally, slight wavenumber shifts were observed at a number of the shared peaks, including around 2850 and 3010 cm^–1^, with PE being slightly blue-shifted. For the proteins, albumin has its primary peak located at 2930 cm^–1^, as opposed to the slightly red-shifted 2935 cm^–1^ peak of histone. Histone was also found to have a relatively weaker aromatic amino acid peak at 3060 cm^–1^ and a more prominent 2875 cm^–1^ shoulder, as compared to albumin. These differences help account for the variety of proteins and lipids found in bacteria, which, in turn, should enable more accurate spectral unmixing.

Looking at the cell wall components, peptidoglycan was found to have an overall spectral shape similar to that of protein, likely due to its amino acid content, but with a less prominent protein-associated peak at 2932 cm^–1^. The overall broader spectral shape is likely due to the glycan backbone of peptidoglycan providing a carbohydrate signal, while various modifications and additions account for the rest. In contrast, the amino sugar was found to have a major peak at 2939 cm^–1^, a smaller peak at 2990 cm^–1^, and a shoulder centered around 2900 cm^–1^. While amino sugars do have one to two −CH_3_ moieties, that level of prevalence is not enough to explain the strength of the 2939 cm^–1^ peak. As such, the sharp peak likely originates from the overlapping vibrational modes of specific −CH bonds, which have been reported for glucose and its derivatives.^41^ That leaves the 2900 and 2990 cm^–1^ spectral features, which likely originate from the methylene and methine groups.^38^ Together, these eight biochemical components are expected to accurately approximate the biochemical composition of most microbial species. This includes species not directly explored in this work, given that they do not have a distinctly different composition that requires inclusion of a specific component, such as can be found with *Mycobacterium* species and mycolic acids.

To account for the unique composition of the lipid-rich *Mycobacterium* species, the three most common variants of mycolic acid, alpha-MA, keto-MA, and methoxy-MA, were also characterized (Figure 4F). All three variations were found to be highly similar to one another, as expected, with major peaks at 2846 and 2881 cm^–1^ corresponding to the symmetric and asymmetric stretching of the methylene group. Additionally, all three have a −CH_3_ shoulder at 2933 cm^–1^ and a small peak around 2993 cm^–1^ that has previously been attributed to the cyclopropane rings characteristic of mycolic acids.^44^ Differences among the three arise primarily from the relative intensities of these features, with alpha-MA exhibiting a stronger cyclopropane signal, which aligns with its structure, and methoxy-MA displaying a more pronounced −CH_3_ shoulder and relatively less asymmetric methylene stretching than the other two.

### Spectral Unmixing and Biochemical Characterization

Representative results of the spectral unmixing for three Gram-positive species, three Gram-negative species, the yeast species, and a *Mycobacterium* species can be found in Figure 5. The results for the remaining species are located in Figure S2. Despite the biochemical differences between the species, average relative fitting errors across all measurements were found to be low. For the 14 species, the average error was found to range from 2.36% (*S. pneumoniae*) to 4.30% (*S. aureus*), with an overall average error of only 2.80%. These results follow the consistent improvement in the fitting accuracy that has been observed from leveraging high-wavenumber spectra, as opposed to fingerprint spectra, for component analysis.^20,45–48^ This improvement is likely due to the lower number of vibrational modes contained in the high-wavenumber region, enabling the representative biomolecules to better approximate the spectral shapes of the more varied components present in the microbial cells.

**Figure 5:**
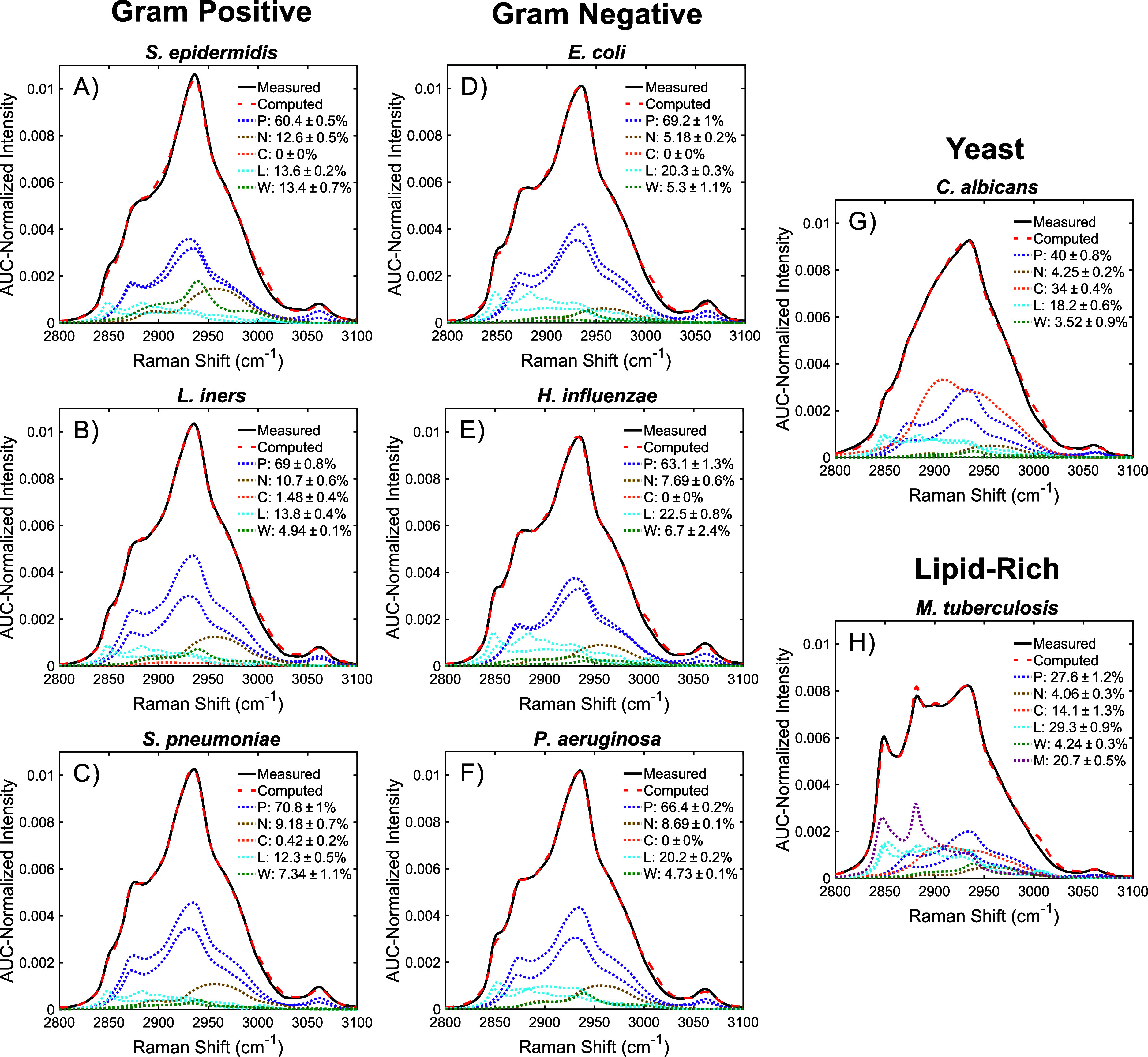
Representative spectral unmixing results for three Gram-positive species (A, B, C), three Gram-negative species (D, E, F), a yeast species (G), and a lipid-rich *Mycobacterium* species (H). Computed spectra (dashed lines) represent the sum of each pure component multiplied by its fractional contribution. Pure components (dotted lines) are grouped into the biochemical categories proteins (P), nucleic acids (N), carbohydrates (C), lipids (L), cell wall components (W), and mycolic acids (M). Percentages and errors reported in each legend are the mean and SEM for the results of the 9 droplet averages.

Looking first across the different cell wall types, the predicted reasons underlying the differences in the yeast and lipid-rich spectra align with the results of the spectral unmixing. *C. albicans* spectra have a much greater fraction of carbohydrate signal than other microbial species, likely due to the glucan used as a component of their cell walls (Figure 5G).^25^ The lipid-rich *Mycobacterium* species, on the other hand, predictably have a significantly higher Raman fraction from lipid species, with the combination of the general lipids and the mycolic acids providing approximately half their total −CH_*x*_ signal. However, it can be seen that the representative lipids used for the unmixing are not completely sufficient for the *Mycobacterium* species, as the computed spectra tended to overestimate the measured signal at the 2880 and 3005 cm^–1^ lipid peaks (Figure 5H). This unideal fitting could also be the cause of the relatively high carbohydrate Raman fraction observed. The carbohydrate signal is often used by the unmixing algorithm to compensate for otherwise poor spectral fits, as it is centered within the −CH_*x*_ band and contains only minimally distinct spectral features. While many *Mycobacterium* species have polysaccharide capsules, it is unlikely that this high carbohydrate Raman fraction is indicative of the underlying composition, though biochemical experiments would be required to validate this.

While less distinct than the differences observed in the yeast and *Mycobacterium* species, clear trends can also be observed between the Gram-positive (Figure 5A-C) and Gram-negative bacteria (Figure 5D-F). Gram-negative species were found to have a significantly higher lipid fraction, with an average of 21.0%, as compared to the Gram-positive species which had an average of only 12.1%. This difference is mirrored by the Raman spectral fraction attributed to the cell wall components, with an average of 5.6% for the Gram-negative species and 11.4% for the Gram-positive species. These trends match the basic physiology of the bacteria, with Gram-negative species having a thinner (2–10 nm) peptidoglycan cell wall surrounded by two lipid bilayer membranes and Gram-positive species having a thicker (20–80 nm) cell wall outside of a single cell membrane.^31^ This relationship can be expressed using the cell wall-to-lipid ratio, for which Gram-positive bacteria had an average value of 0.99 compared to the 0.26 of Gram-negative bacteria.

Using this ratio, known physiological traits of individual species can also be observed. *L. iners* is known to have a thin cell wall which leads to indeterminate Gram staining despite having a Gram-positive cell wall structure.^49^ Matching this, the average cell wall-to-lipid ratio for *L. iners* was found to be 0.36, which is much closer to the Gram-negative ratios. Another species known to be gram-indeterminate due to its thin cell wall is *G. vaginalis*.^50^ Despite the relatively high cell wall contribution from the spectral unmixing, the average cell wall-to-lipid ratio for *G. vaginalis* was found to be 0.79, with only one other Gram-positive species, *S. pneumoniae*, having a lower ratio. This indicates that high-wavenumber measurements are broadly sensitive to the relative cell wall thickness of bacterial species.

While *S. pneumoniae* is known to have a thick cell wall, seemingly contradicting the results obtained herein, the peptidoglycan portion of it has been reported to only be ∼ 15 nm thick, which may explain its relatively low cell wall-to-lipid ratio of 0.60.^51^ The rest of the cell wall is composed of teichoic acids, bacterial copolymers found in the cell walls of most Gram-positive species, which consist of long chains of glycerol phosphate or ribitol phosphate and sugar moieties.^52^ While teichoic acids could be included as a standalone component, their structure implies that they would overlap spectrally with both the lipid and carbohydrate groups. Moreover, as including more component spectra in the spectral unmixing analysis increases the risk of cross-talk and overfitting, there is a strong chance that the inclusion of teichoic acids, or any other additional components, would decrease the overall performance.

### Raman Dry Mass

Relative dry mass composition is a useful analytical metric for assessing the physiology and metabolism of bacteria under different growth conditions.^34,35^ However, current biochemical techniques to obtain dry mass are multifaceted and cumbersome, leading to only a small number of species that have been fully characterized. While the reported Raman spectral fractions correlate to physiological differences, they do not directly match microbial compositions due to the different classes of biomolecules having different Raman cross sections. To address this, this work utilizes a newly described method to convert the Raman contributions from each unmixed spectrum into estimates of the relative Raman dry masses.^53^ Out of the 14 microbial species utilized in this work, only the dry mass values for *E. coli* have been previously reported.^34,35,37^ From the handful of studies that have characterized *E. coli*, expected ranges for the relative dry masses of protein, nucleic acid, carbohydrate, lipid, and cell wall were obtained, to which the Raman dry mass values were compared (Table 2). The relative Raman dry mass values for all 14 microbial species can be found in Table S3.

**Table 2:** Raman Dry Mass Values Determined for *E. coli* Compared to the Reported Dry Mass Composition

	**Reported** ^a^ **Dry Mass**	**Raman Dry Mass**
**Protein**	50–65%	63.2 ± 0.83%
**Nucleic Acid**	13–27%	18.3 ± 0.77%
**Carbohydrate**	∼2.5%	0%
**Lipid**	9–15%	12.9 ± 0.16%
**Cell Wall**	3–10%	5.64 ± 1.23%

a Compiled from refs 34, 35, and 37 .

Overall, the Raman dry mass values for *E. coli* were found to fall within the reported ranges, indicating the accuracy of the conversion. Unfortunately, due to the difficulties of using traditional techniques to extract, purify, and quantify biochemical components, the reported dry mass values for *E. coli* vary significantly, even when using the same strain and growth conditions.^34,35^ While this variability makes direct comparisons difficult, the effectiveness of the Raman dry mass approach can still be observed. Most notably, the Raman intensity fractions attributed to both nucleic acids (5.18%) and lipids (20.3%) fall significantly outside their respective dry mass ranges, while their respective Raman dry mass estimates fall solidly within the reported ranges.

While the values for nucleic acids, lipids, and the cell wall are close to the center of their ranges, both the protein and carbohydrate levels are near their reported extremes. For the carbohydrate fraction, the Raman dry mass fell below the expected values, with the original spectral unmixing yielding zero glycogen contribution. While the culture conditions may have led to a minimal store of glycogen and other carbohydrates within the bacteria, this result is more likely due to a limitation of the spectral unmixing itself when dealing with low concentrations of these compounds. Carbohydrates only comprise around 2.5% of the dry mass of *E. coli*. Thus, the overall Raman contribution may be too small to resolve, given the lack of distinct carbohydrate spectral features. Some of these mischaracterized signals may have been misattributed to protein, as the Raman dry mass for proteins was found to be close the maximum reported value of 65%. Additionally, the use of peptidoglycan from a Gram-positive species may have influenced the results of the spectral unmixing, resulting in a greater Raman fraction of protein due to both peptidoglycan and protein containing spectral features associated with amino acids. However, the inclusion of amino sugars as a separate component in the spectral unmixing appears to have helped mitigate this error. This is evidenced by the Raman dry mass of the *E. coli* cell wall being in the middle of the reported range, as well as all three Gram-negative bacteria having similar cell wall spectral contributions. Therefore, it can be concluded that the proposed Raman dry mass approximation yields biologically accurate results, showcasing the ability of high-wavenumber Raman spectroscopy to biochemically characterize microbial samples. Future studies directly comparing Raman dry mass against traditional biochemical techniques will be required to fully determine the accuracy of using high-wavenumber Raman spectroscopy to characterize the biochemical composition of bacteria, cells, and other biological materials.

## Conclusions

This study aimed to investigate the capability of using high-wavenumber Raman spectroscopy to both identify and characterize microbial species. To achieve this, high-wavenumber Raman spectra were collected for 14 different species, including Gram-positive bacteria, Gram-negative bacteria, gram-indeterminate bacteria, and yeast. These spectra were used to train a PLS-SVM decision tree capable of multiple levels of classification. Distinct spectral differences observed between the different species resulted in high classification accuracies when determining cell wall type (100%), genus (98.9%), and species (97.4%). Additionally, this work demonstrated that the −CH_*x*_ band can be spectrally decomposed into representative component spectra, taking advantage of the differences in spectral shape between proteins, nucleic acids, carbohydrates, lipids, and specific cell wall components. The resulting Raman spectral fractions were found to closely match expected physiological differences. Strong differences were observed in the cell wall-to-lipid ratio between Gram-positive (0.99) and Gram-negative (0.26) bacteria, which approximate cell envelope compositional differences. Finally, a newly described method of converting high-wavenumber Raman contributions into estimates of the relative dry mass composition was used, with results for the highly characterized *E. coli* falling within previously reported ranges. Overall, this work demonstrates that high-wavenumber Raman spectroscopy is a feature-rich, low-background technique capable of both differentiating between different microbial species and nondestructively assessing their biomolecular composition.

## Supplementary Material



## Notes

Additional experimental details including an example of the difference in Raman-to-background ratios for fingerprint and high-wavenumber measurements of bacteria, specific information on the species and strains used in the work, the parameters used in the Raman dry mass calculations for each class of biomolecule, additional representative spectral unmixing results, and the numerical results of the Raman dry mass compositions for all utilized species (PDF)

## References

[1] Rajapaksha P., Elbourne A., Gangadoo S., Brown R., Cozzolino D., Chapman J. (2019). A Review of Methods
for the Detection of Pathogenic
Microorganisms. Analyst.

[2] Gupta S., Kakkar V. (2018). Recent Technological
Advancements in Tuberculosis Diagnostics
– A Review. Biosens Bioelectron.

[3] Rentschler S., Kaiser L., Deigner H.-P. (2021). Emerging
Options for the Diagnosis
of Bacterial Infections and the Characterization of Antimicrobial
Resistance. Int. J. Mol. Sci..

[4] Diallo K., Feteh V. F., Ibe L., Antonio M., Caugant D. A., du Plessis M., Deghmane A.-E., Feavers I. M., Fernandez K., Fox L. M., Rodrigues C. M. C., Ronveaux O., Taha M.-K., Wang X., Brueggemann A. B., Maiden M. C. J., Harrison O. B. (2021). Molecular
Diagnostic Assays for the Detection of Common Bacterial Meningitis
Pathogens: A Narrative Review. EBioMedicine.

[5] Avershina E., Khezri A., Ahmad R. (2023). Clinical Diagnostics
of Bacterial
Infections and Their Resistance to Antibiotics—Current State
and Whole Genome Sequencing Implementation Perspectives. Antibiotics.

[6] Locke A. K., Zaki F. R., Fitzgerald S. T., Sudhir K., Monroy G. L., Choi H., Won J., Mahadevan-Jansen A., Boppart S. A. (2022). Differentiation of Otitis Media-Causing
Bacteria and
Biofilms via Raman Spectroscopy and Optical Coherence Tomography. Front Cell Infect Microbiol.

[7] Kloß S., Kampe B., Sachse S., Rösch P., Straube E., Pfister W., Kiehntopf M., Popp J. (2013). Culture Independent Raman Spectroscopic Identification of Urinary
Tract Infection Pathogens: A Proof of Principle Study. Anal. Chem..

[8] Harz M., Kiehntopf M., Stöckel S., Rösch P., Straube E., Deufel T., Popp J. (2009). Direct Analysis of
Clinical Relevant Single Bacterial Cells from Cerebrospinal Fluid
during Bacterial Meningitis by Means of Micro-Raman Spectroscopy. J. Biophotonics.

[9] Kumar S., Gopinathan R., Chandra G. K., Umapathy S., Saini D. K. (2020). Rapid Detection
of Bacterial Infection and Viability Assessment with High Specificity
and Sensitivity Using Raman Microspectroscopy. Anal Bioanal Chem..

[10] Ayala O. D., Doster R. S., Manning S. D., O’Brien C. M., Aronoff D. M., Gaddy J. A., Mahadevan-Jansen A. (2019). Raman Microspectroscopy
Differentiates Perinatal Pathogens on Ex Vivo Infected Human Fetal
Membrane Tissues. J. Biophotonics.

[11] Ho C.-S., Jean N., Hogan C. A., Blackmon L., Jeffrey S. S., Holodniy M., Banaei N., Saleh A. A. E., Ermon S., Dionne J. (2019). Rapid Identification
of Pathogenic Bacteria Using Raman
Spectroscopy and Deep Learning. Nat. Commun..

[12] Locke A., Fitzgerald S., Mahadevan-Jansen A. (2020). Advances in Optical Detection of
Human-Associated Pathogenic Bacteria. Molecules.

[13] Pahlow S., Meisel S., Cialla-May D., Weber K., Rösch P., Popp J. (2015). Isolation and Identification
of Bacteria by Means of Raman Spectroscopy. Adv. Drug Deliv Rev..

[14] Wang L., Tang J.-W., Li F., Usman M., Wu C.-Y., Liu Q.-H., Kang H.-Q., Liu W., Gu B. (2022). Identification
of Bacterial Pathogens at Genus and Species Levels through Combination
of Raman Spectrometry and Deep-Learning Algorithms. Microbiol Spectr.

[15] Shen H., Rösch P., Thieme L., Pletz M. W., Popp J. (2023). Comparison
of Bacteria in Different Metabolic States by Micro-Raman Spectroscopy. J. Mol. Struct..

[16] Contreras J., Mostafapour S., Popp J., Bocklitz T. (2024). Siamese Networks for
Clinically Relevant Bacteria Classification Based on Raman Spectroscopy. Molecules.

[17] Kusić D., Kampe B., Ramoji A., Neugebauer U., Rösch P., Popp J. (2015). Raman Spectroscopic Differentiation
of Planktonic Bacteria and Biofilms. Anal Bioanal
Chem..

[18] Mo J., Zheng W., Low J. J. H., Ng J., Ilancheran A., Huang Z. (2009). High Wavenumber Raman Spectroscopy for in Vivo Detection of Cervical
Dysplasia. Anal. Chem..

[19] Lin K., Zheng W., Lim C. M., Huang Z. (2017). Real-Time *In
Vivo* Diagnosis of Nasopharyngeal Carcinoma Using Rapid Fiber-Optic
Raman Spectroscopy. Theranostics.

[20] Haugen E.
J., Locke A. K., Dao L. H., Walter A. B., Rasiah P. K., Baba J. S., Buendia M. A., Correa H., Hiremath G., Mahadevan-Jansen A. (2025). Biochemical
Detection of Pediatric Eosinophilic Esophagitis
Using High Wavenumber Raman Endoscopy and Stimulated Raman Microscopy. Sci. Rep.

[21] Lee K. S., Landry Z., Pereira F. C., Wagner M., Berry D., Huang W. E., Taylor G. T., Kneipp J., Popp J., Zhang M., Cheng J.-X., Stocker R. (2021). Raman Microspectroscopy
for Microbiology. Nature Reviews Methods Primers.

[22] Kennedy A. D., Otto M., Braughton K. R., Whitney A. R., Chen L., Mathema B., Mediavilla J. R., Byrne K. A., Parkins L. D., Tenover F. C., Kreiswirth B. N., Musser J. M., DeLeo F. R. (2008). Epidemic
Community-Associated Methicillin-Resistant *Staphylococcus
Aureus*: Recent Clonal Expansion and Diversification. Proc. Natl. Acad. Sci. U. S. A..

[23] Kabir M. A., Hussain M. A., Ahmad Z. (2012). *Candida
Albicans*: A Model Organism for Studying Fungal Pathogens. ISRN Microbiol.

[24] Lieber C. A., Mahadevan-Jansen A. (2003). Automated
Method for Subtraction of Fluorescence from
Biological Raman Spectra. Appl. Spectrosc..

[25] Gow N. A. R., Lenardon M. D. (2023). Architecture
of the Dynamic Fungal Cell Wall. Nat. Rev. Microbiol.

[26] Jackson M. (2014). The Mycobacterial
Cell Envelope--Lipids. Cold Spring Harb Perspect
Med..

[27] Stojkova P., Spidlova P., Stulik J. (2019). Nucleoid-Associated
Protein HU: A
Lilliputian in Gene Regulation of Bacterial Virulence. Front Cell Infect Microbiol.

[28] Dorman C. J. (2015). Function
of Nucleoid-Associated Proteins in Chromosome Structuring and Transcriptional
Regulation. Microb Physiol.

[29] Sohlenkamp C., Geiger O. (2016). Bacterial Membrane
Lipids: Diversity in Structures
and Pathways. FEMS Microbiol Rev..

[30] Lu F.-K., Basu S., Igras V., Hoang M. P., Ji M., Fu D., Holtom G. R., Neel V. A., Freudiger C. W., Fisher D. E., Xie X. S. (2015). Label-Free
DNA Imaging in Vivo with
Stimulated Raman Scattering Microscopy. Proc.
Natl. Acad. Sci. U. S. A..

[31] Rohde, M. The Gram-Positive Bacterial Cell Wall. Microbiol Spectr 2019, 7 (3). 10.1128/microbiolspec.GPP3-0044-2018. PMC11086966 31124431

[32] Keresztury, G. Raman Spectroscopy: Theory. In Handbook of Vibrational Spectroscopy; Chalmers, J. M. , Ed.; Wiley, 2001. 10.1002/0470027320.s0109.

[33] Aggarwal R. L., Farrar L. W., Saikin S. K., Aspuru-Guzik A., Stopa M., Polla D. L. (2011). Measurement of the Absolute Raman
Cross Section of the Optical Phonon in Silicon. Solid State Commun..

[34] Beck A., Hunt K., Carlson R. (2018). Measuring
Cellular Biomass Composition
for Computational Biology Applications. Processes.

[35] Simensen V., Schulz C., Karlsen E., Bråtelund S., Burgos I., Thorfinnsdottir L. B., García-Calvo L., Bruheim P., Almaas E. (2022). Experimental Determination
of Escherichia
Coli Biomass Composition for Constraint-Based Metabolic Modeling. PLoS One.

[36] Portevin D., Sukumar S., Coscolla M., Shui G., Li B., Guan X. L., Bendt A. K., Young D., Gagneux S., Wenk M. R. (2014). Lipidomics and Genomics
of *Mycobacterium Tuberculosis* Reveal Lineage-specific
Trends in Mycolic Acid Biosynthesis. Microbiologyopen.

[37] Schönheit P., Buckel W., Martin W. F. (2016). On the Origin of Heterotrophy. Trends Microbiol.

[38] Talari A. C. S., Movasaghi Z., Rehman S., Rehman I. ur. (2015). Raman Spectroscopy
of Biological Tissues. Appl. Spectrosc Rev..

[39] Howell N. K., Arteaga G., Nakai S., Li-Chan E. C. Y. (1999). Raman Spectral
Analysis in the C–H Stretching Region of Proteins and Amino
Acids for Investigation of Hydrophobic Interactions. J. Agric. Food Chem..

[40] Czamara K., Majzner K., Pacia M. Z., Kochan K., Kaczor A., Baranska M. (2015). Raman Spectroscopy
of Lipids: A Review. J. Raman Spectrosc..

[41] Wiercigroch E., Szafraniec E., Czamara K., Pacia M. Z., Majzner K., Kochan K., Kaczor A., Baranska M., Malek K. (2017). Raman and
Infrared Spectroscopy of Carbohydrates: A Review. Spectrochim Acta A Mol. Biomol Spectrosc.

[42] Takahashi, F. ; Abe, S. Decision-Tree-Based Multiclass Support Vector Machines. In Proceedings of the 9th International Conference on Neural Information Processing, 2002. ICONIP ’02.; Nanyang Technol. Univ, 2002; Vol. 3, pp 1418–1422. 10.1109/ICONIP.2002.1202854.

[43] Koljenović S., Bakker Schut T. C., Wolthuis R., de Jong B., Santos L., Caspers P. J., Kros J. M., Puppels G. J. (2005). Tissue Characterization
Using High Wave Number Raman Spectroscopy. J.
Biomed Opt.

[44] Kochan K., Peng H., Gwee E. S. H., Izgorodina E., Haritos V., Wood B. R. (2019). Raman Spectroscopy as a Tool for
Tracking Cyclopropane Fatty Acids in Genetically Engineered *Saccharomyces Cerevisiae*. Analyst.

[45] Walter A.
B., Whitehead L., Taylor A. L., Locke A. K. (2025). High-Wavenumber
Raman Spectroscopy for the Detection of *Mycobacterium Tuberculosis* in Saliva. Sensors & Diagnostics.

[46] Daniel A., Prakasarao A., Ganesan S. (2018). Near-Infrared Raman Spectroscopy
for Estimating Biochemical Changes Associated with Different Pathological
Conditions of Cervix. Spectrochim Acta A Mol.
Biomol Spectrosc.

[47] O’Brien C. M., Vargis E., Rudin A., Slaughter J. C., Thomas G., Newton J. M., Reese J., Bennett K. A., Mahadevan-Jansen A. (2018). In Vivo Raman Spectroscopy for Biochemical Monitoring
of the Human Cervix throughout Pregnancy. Am.
J. Obstet Gynecol.

[48] Spurlin E. E., Belcher K., Shofu F., Esteves J. A. E., Jimenez P. T., O’Brien C. M. (2025). In Vivo
Raman Spectroscopy Reveals Biochemical Changes
in the Human Cervix with Pregnancy Establishment. npj Women’s Health.

[49] Kim H., Kim T., Kang J., Kim Y., Kim H. (2020). Is Lactobacillus
Gram-Positive?
A Case Study of Lactobacillus Iners. Microorganisms.

[50] Sadhu K., Domingue P. A. G., Chow A. W., Nelligan J., Cheng N., Costerton J. W. (1989). Gardnerella
Vaginalis Has a Gram-Positive Cell-Wall
Ultrastructure and Lacks Classical Cell-Wall Lipopolysaccharide. J. Med. Microbiol.

[51] Nguyen, M. ; Bauda, E. ; Boyat, C. ; Laguri, C. ; Freton, C. ; Chouquet, A. ; Gallet, B. ; Baudoin, M. ; Wong, Y.-S. ; Grangeasse, C. ; Moriscot, C. ; Durmort, C. ; Zapun, A. ; Morlot, C. Teichoic Acids in the Periplasm and Cell Envelope of Streptococcus Pneumoniae. Elife 2025, 14. 10.7554/eLife.105132. PMC12017771 40265569

[52] Brown S., Santa Maria J. P., Walker S. (2013). Wall Teichoic Acids of Gram-Positive
Bacteria. Annu. Rev. Microbiol..

[53] Haugen, E. J. ; Walter, A. B. ; Estes, B. J. ; Locke, A. K. ; Mahadevan-Jansen, A. Development of Biochemical Standards for High Wavenumber Raman Microspectroscopy and Fiber-Optic Raman Applications (Conference Presentation). In Design and Quality for Biomedical Technologies XVIII; Vargas, G. , Ed.; SPIE, 2025; Vol. PC13308, p 11. 10.1117/12.3040385.

