# Supplementary material for: High-Wavenumber Raman Spectroscopy for the Identification and Biochemical Characterization of Microbial Species
Source: Anal Chem. 2025 Dec 17;97(51):28270–80. doi: 10.1021/acs.analchem.5c05031 (PMC12756854; doi:10.1021/acs.analchem.5c05031)
Supplement: Supplementary file 1 [file ac5c05031_si_001.pdf]

Supporting Information

High-wavenumber Raman spectroscopy for the identification and biochemical characterization of microbial species

Alec B. Walter[1,2], Ezekiel Haugen[1,2], Anna S. Rourke-Funderburg[1,2], Andrea K. Locke[1,2,3,]*

[1]Vanderbilt Biophotonics Center, Vanderbilt University, Nashville, TN, 37240, USA

[2]Department of Biomedical Engineering, Vanderbilt University, Nashville, TN, 37240, USA

[3]Department of Chemistry, Vanderbilt University, Nashville, TN, 37240, USA

*Andrea K. Locke, E-mail: andrea.locke@vanderbilt.edu

Table of Contents

Figure S1. Comparison of the Raman-to-background ratios for fingerprint and high-wavenumber measurements of bacteria **S2**

Table S1. Microbial species and strains used in this work **S3**

Table S2. Estimated Raman dry mass parameters for each biochemical class. **S4**

Figure S2. Representative spectral unmixing results for the remaining Gram-positive and Mycobacterium species. **S5**

Table S3. Raman dry mass compositions for the 14 microbial species **S6**

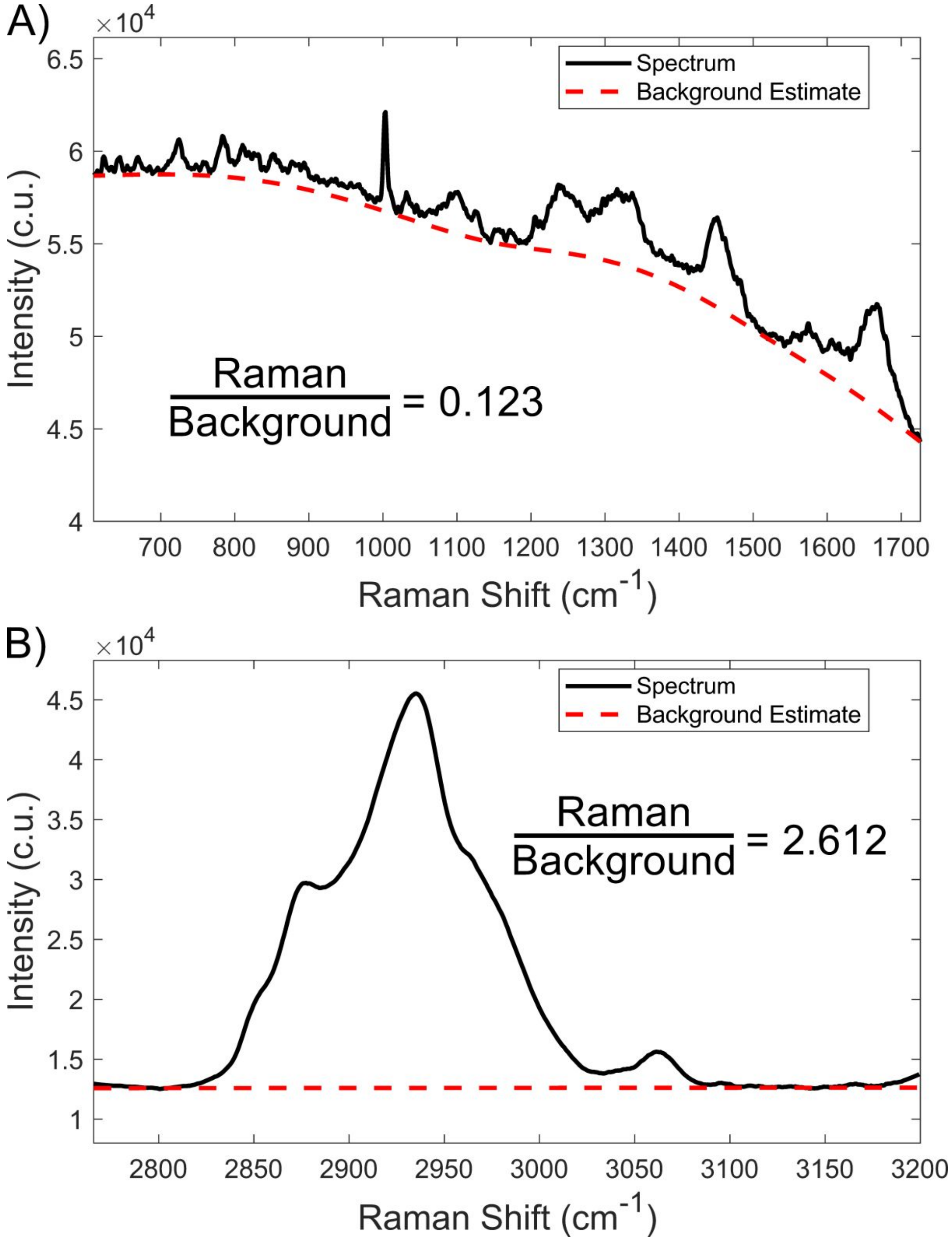

Figure S1. Comparison of the Raman-to-background ratios for *S. pneumoniae* within the (A) fingerprint region and the (B) high-wavenumber region. Spectra were acquired in an alternating fashion from the same spot. Fingerprint spectra were measured using approximately 40 mW of power at the sample and a total integration time of 20 seconds (2-second exposure, 10 accumulations). The reported Raman-to-background ratios are the maximum value of the ratio of the background subtracted Raman spectra to the estimated background autofluorescence.

Table S1. Microbial species and strains used in this work.

Species	Strain	Growth Media
Staphylococcus aureus	USA300 LAC	TSB
Staphylococcus epidermidis	FDA Strain PCI 1200 (ATCC 12228)	TSB
Gardnerella vaginalis	594 (ATCC 14018)	NYCIII
Lactobacillus crispatus	VPI 7635 (ATCC 33197)	MRS
Lactobacillus iners	ATCC 55195	NYCIII
Streptococcus mutans	UA159 (ATCC 700610)	BHI
Streptococcus agalactiae	NCTC 8181 (ATCC 13813)	BHI
Streptococcus pneumoniae	ATCC 6301	BHI
Escherichia coli	NCTC 9001 (ATCC 11775)	TSB
Pseudomonas aeruginosa	ATCC 14203	TSB
Haemophilus influenzae	L-378 (ATCC 49766)	sBHI
Mycobacterium tuberculosis	H37Ra (ATCC 25177)	Middlebrook
Mycobacterium bovis	BCG Pasteur (ATCC 35734)	Middlebrook
Candida albicans	NCCLS 11 (ATCC 90028)	SDB

TSB: tryptic soy broth, MRS: de Man–Rogosa–Sharpe, BHI: brain heart infusion, sBHI: supplemented BHI

Table S2. The approximate number of $-CH_x$ bond (B) and molecular weight for the monomers of each biochemical class used for the Raman dry mass approximation.

Biochemical Class	B	MW (g/mol)
Protein	3.9	108.0
Nucleic Acid	5	479.5
Carbohydrate	6	180.2
Lipid	37	719.0
Peptidoglycan	15	501.0
Amino Sugar	8	257.2
Mycolic Acid	79	1166.1

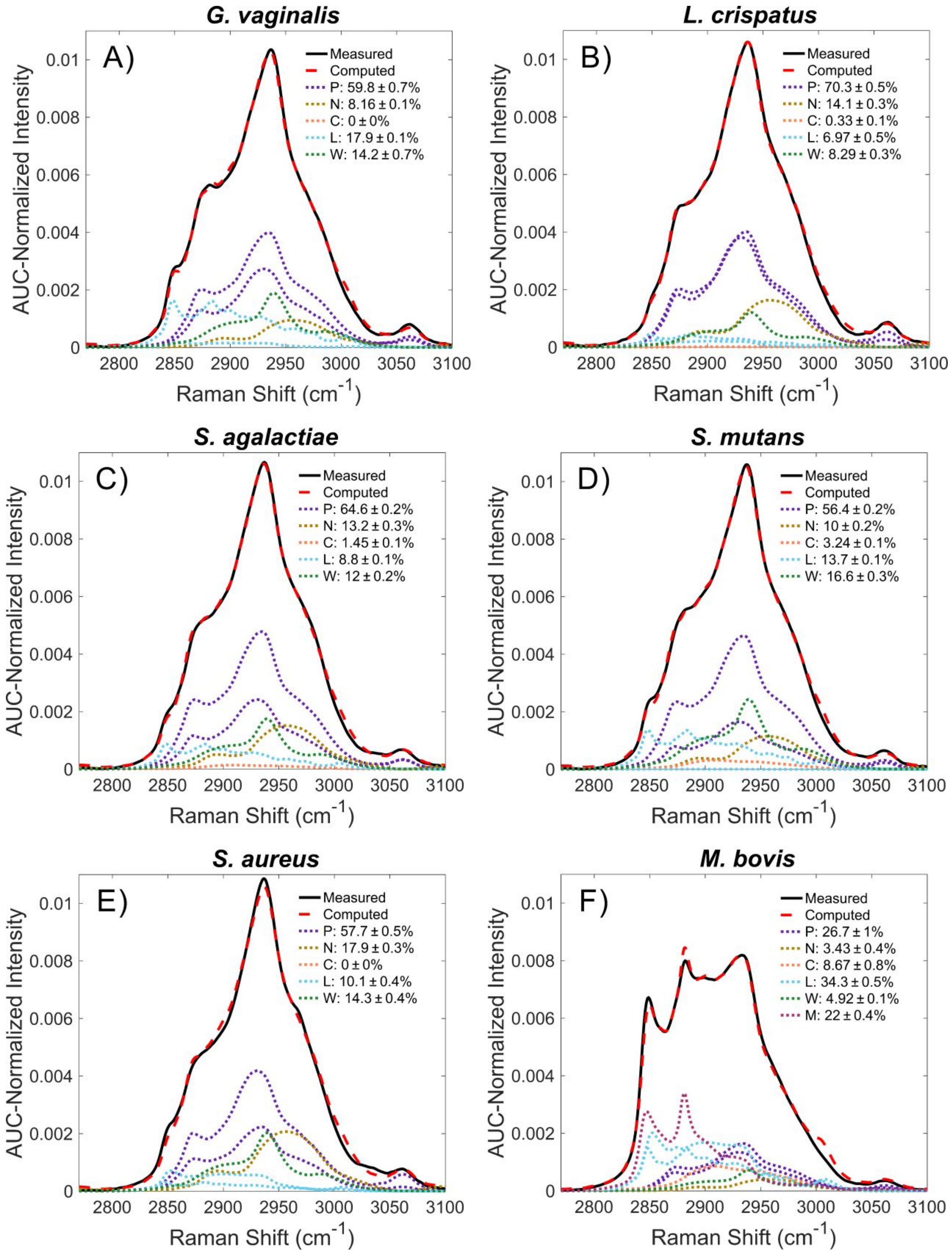

Figure S2. Representative spectral unmixing results for the remaining five Gram-positive species (A, B, C, D, E) and *Mycobacterium* species (F). Computed spectra (dashed lines) represent the sum of each pure component multiplied by its fractional contribution. Pure components (dotted lines) are grouped into the biochemical categories proteins (P), nucleic acids (N), carbohydrates (C), lipids (L), cell wall components (W), and mycolic acids (M). Percentages and errors reported in each legend are the mean and SEM for the results of the 9 droplet measurements.

Table S3. Raman dry mass compositions for the 14 microbial species. Values are reported as mean and (SEM) for the 9 droplet samples.

	Protein (%)	Nucleic Acid (%)	Carbohydrate (%)	Lipid (%)	Cell Wall (%)	Mycolic Acid (%)
S. aureus	43.1 (1.68)	39.2 (2.27)	0 (0)	5.50 (0.35)	12.2 (0.49)	0 (0)
S. epidermidis	48.2 (0.76)	30.5 (1.71)	0 (0)	8.06 (0.51)	13.2 (1.04)	0 (0)
G. vaginalis	51.1 (0.75)	24.1 (0.35)	0 (0)	10.72 (0.11)	14.1 (0.68)	0 (0)
L. crispatus	53.6 (1.01)	34.1 (1.67)	1.29 (0.73)	3.71 (0.24)	7.34 (0.27)	0 (0)
L. iners	56.6 (1.04)	29.0 (1.21)	1.53 (0.36)	7.92 (0.24)	4.92 (0.26)	0 (0)
S. mutans	46.7 (0.39)	25.9 (0.84)	3.10 (0.14)	8.24 (0.20)	16.1 (0.25)	0 (0)
S. agalactiae	50.6 (0.99)	31.1 (1.95)	1.87 (0.39)	4.88 (0.14)	11.5 (0.49)	0 (0)
S. pneumoniae	59.3 (1.36)	25.8 (1.25)	0.31 (0.16)	7.26 (0.37)	7.32 (1.11)	0 (0)
E. coli	63.2 (0.83)	18.3 (0.77)	0 (0)	12.9 (0.16)	5.64 (1.23)	0 (0)
P. aeruginosa	57.3 (0.16)	25.7 (0.17)	0 (0)	12.2 (0.12)	4.78 (0.09)	0 (0)
H. influenzae	55.7 (0.97)	23.1 (1.47)	0 (0)	13.9 (0.47)	7.24 (2.56)	0 (0)
M. tuberculosis	29.5 (1.17)	15.0 (1.07)	16.4 (1.64)	22.0 (0.77)	5.29 (0.37)	11.8 (0.25)
M. bovis	29.9 (1.15)	13.1 (1.37)	10.6 (1.03)	26.9 (0.41)	6.37 (0.10)	13.1 (0.36)
C. albicans	36.9 (0.84)	13.5 (0.61)	33.9 (0.41)	11.8 (0.46)	3.79 (0.96)	0 (0)
